# Sarcomatoid carcinoma of the penis: Clinicopathologic features

**DOI:** 10.4103/0970-1591.40630

**Published:** 2008

**Authors:** R. Ranganath, Shirley Sunder Singh, B. Sateeshan

**Affiliations:** Division of Surgical Oncology, Cancer Institute (WIA), Adyar, Chennai, Tamil Nadu, India; 1 Department of Pathology, Cancer Institute (WIA), Adyar, Chennai, Tamil Nadu, India

**Keywords:** Clinical features, immunopathology, sarcomatoid carcinoma

## Abstract

Sarcomatoid carcinomas are biphasic tumors, which can occur at any site in the human body. Very few cases have been reported in the literature as arising from the penis. A few studies consider these tumors as a variant of squamous cell carcinoma or a metaplastic differentiation of the mesenchyme. Their clinical behavior is aggressive with both blood borne and lymphatic metastases. Treatment is by surgical excision, and dissected lymph nodes have shown both epithelial and sarcomatous components. We report a 50-year-old gentleman, with a sarcomatoid carcinoma of the penis, which was confirmed immunopathologically. The rarity of this entity makes it a clinicopathologic curiosity.

## INTRODUCTION

The incidence of penile cancer in the Indian subcontinent is 1.8/100,000 population.[1] Almost 95% of these tumors are squamous cell carcinomas.

An uncommon variant is sarcomatoid carcinoma, which has also been called as spindle cell carcinoma, metaplastic carcinoma, or biphasic squamous cell carcinoma. Most pathologists today accept that carcinosarcoma is a tumor which originates from an epithelial cell. These tumors express both epithelial as well as mesenchymal antigens, when tested by immunohistochemistry.

We report a patient of penile sarcomatoid carcinoma treated in our Institute, with the necessary immuno-pathologic correlation.

## CASE REPORT

A 60 years old male presented with complaints of an ulcerated lesion on the glans penis of 3 months duration. Clinical examination revealed a 3 × 2 cm ulcerated tumor involving the dorsal aspect of the glans penis. The penile shaft was not involved by the tumor. The urethral meatus was free. There were insignificant 0.5 cm nodes in both inguinal regions.

Histopathologic examination revealed the tumor to be a sarcomatoid carcinoma. A chest radiograph did not reveal any abnormalities.

The patient underwent a partial amputation and had an uneventful postoperative recovery. He has completed 6 months of follow-up and is doing well.

**Figure 1:**
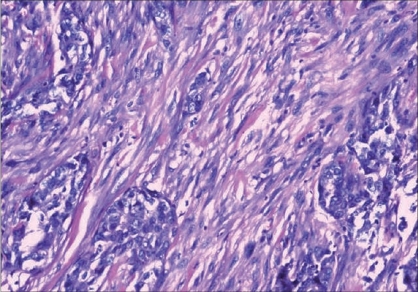
Nests and islands of malignant squamous cells admixed with sarcomatous component

**Figure 2:**
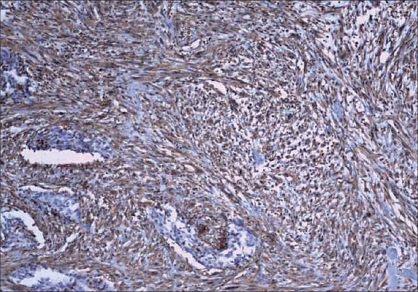
Sarcomatous areas showing immunoreactivity to vimentin

Multiple sections were taken from the primary tumor for histopathologic examination. Paraffin embedded, hematoxylin, and eosin stained slides were first used to establish the tumor morphology and initial histopathological tumor typing. The microscopic picture showed fascicles and sheets of spindle-shaped cells with moderate eosinophilic cytoplasm, admixed with areas of atypical squamous epithelial cells [Figure 1]. Myxoid change and osseous metaplasia were also noted. Immunohistochemistry showed positivity of the epithelial cells for epithelial membrane antigen (EMA) and keratin. The sarcomatous area was diffusely positive for vimentin [Figure 2] and also for keratin. The tumor cells were negative for smooth muscle actin, desmin, and S-100.

A final diagnosis of nonmetastatic sarcomatoid carcinoma (undifferentiated) was made.

## DISCUSSION

Sarcomatoid carcinoma of the penis is a rare variant of penile cancer, representing only 1-2% of all penile carcinomas. Most authors consider this tumor to be a variant of squamous cell carcinoma with a poor prognosis.[2]

The microscopic diagnosis of a sarcomatoid carcinoma can prove to be a challenge. In its classic appearance, the tumor consists of a biphasic pattern with areas of pleomorphic spindle cells admixed with a squamous cell carcinoma component.[3] The mesenchymal component of the tumor is often found in the deeper tumor layers. If light microscopy shows only a sarcomatoid pattern, the demonstration of keratin filaments by immunohistochemistry will give us the correct diagnosis.[3]

The differential diagnosis of carcinosarcoma includes leiomyosarcoma, angiosarcoma, amelanotic melanoma among others.[4]

The exact histogenesis of this tumor is still controversial. Most authors believe that the sarcomatoid component develops from the carcinomatous areas by dedifferentiation or more precisely by a premature block in differentiation toward a squamous phenotype.[5] Based on this theory, the two components are considered to originate from the same stem cell. This theory is also supported by an ultrastructural study in which epithelial elements were recognized within the spindle-shaped cells.
